# Problematic White-Tailed Deer Information in Rochlin et al. (2022) Regarding Past and Future Tick (*Amblyomma americanum*, Acari: Ixodidae) Distributions

**DOI:** 10.1093/jme/tjac090

**Published:** 2022-07-08

**Authors:** G Kent Webb

**Affiliations:** School of Information Systems and Technology, College of Business, San Jose State University, One Washington Square, San Jose, CA 95192, USA

**Keywords:** white-tailed deer population, lone star tick, distribution, climate change, land use

## Abstract

Figure 3 in this *Journal of Medical Entomology* article is central to the authors’ warning about an exploding white-tailed deer population but conflicts in important aspects with the relevant deer research. Among other problems, it shows a 60% increase in the white-tailed deer density from 1500 to 2020 when the research consensus is that the population is about the same. It shows an exploding population from 2000 to 2020 without supporting data when the population peaked around the year 2000 according to evidence-based research.

Figure 3 in this *Journal of Medical Entomology* article (Rochlin et al., 2022, p. 415) is central to the author’s warning about an exploding white-tailed deer population but conflicts in important aspects with the relevant deer research. Figure 1 illustrates the problems. For comparison, the white-tailed deer information in the figure is all indexed to 1 for the year 2000. The figure shows the white-tailed deer density (blue boxes) from Rochlin et al. (2022) and the Missouri deer population (green line) from Pierce et al. (2011) that was cited as a data source by Rochlin et al. (2022).

**Fig. 1.**
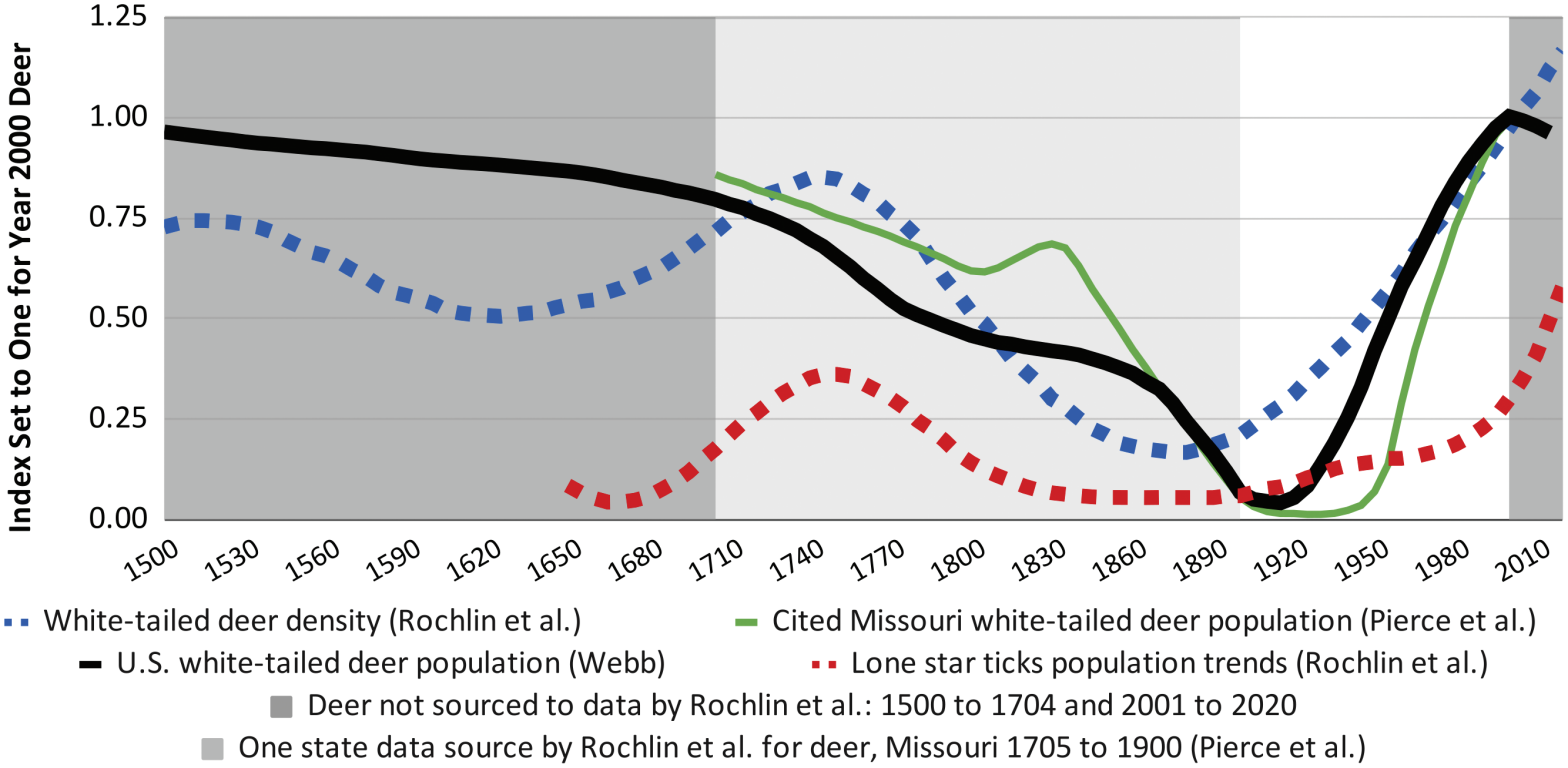
Deer density (blue boxes) in Rochlin et al. (2022) compared to their cited population for Missouri (green line) from Pierce et al. (2011) and conflicting research for the U.S. deer population (black line) from Webb (2018). Deer data is indexed to 1.00 in the year 2000 to allow for comparison of different deer measurements. Tick population trends (red boxes) from Rochlin et al (2022). Dark gray areas, 1500 to 1704 and 2001 to 2020 indicate no graphical or numerical data were cited for deer by Rochlin et al. (2022). The light gray area indicates only data from the state of Missouri is cited for the period 1705 to 1900.

The estimate for the U.S. white-tailed deer population (black line) from Webb (2018) was based on a survey of the literature and thousands of sources supporting the deer population estimates from the year 2000 to 2020. This paper is available on ResearchGate.Net where there is a link to the data sources. Also included in Figure 1 are the tick population trends (red boxes) from Rochlin et al. (2022). Gray areas show how well their deer information was sourced. Graphical information is assumed to be data.

As detailed below, the most significant problems are that Figure 3 in Rochlin et al. (2022) shows: 1) a 60% increase in the white-tailed deer density from 1500 to 2020 when the consensus is that the white-tailed deer population is about the same and their range has expanded so the density may be lower; 2) that the deer population has experienced a dramatic increase since year 2000 when other research shows the population peaked in the year 2000; and 3) that their deer density (blue boxes) which match so closely with their tick populations trends (red boxes) does not match so closely with the other deer research.

The consensus view of the white-tailed deer population is summarized by the University of Florida (2000): “experts believe the population of [white-tailed] deer in the United States is about equal to what it was before Europeans arrived, with somewhere between 24 million and 34 million nationwide.” This estimate can be found in the most cited research on the precolonial North American white-tailed deer population, McCabe and McCabe (1984).

The black line (Webb, 2018) in Figure 1 follows the pattern of VerCauteren (2003) that is consistent with McCabe and McCabe (1984) for the years 1500 to 2000. There were a small number of white-tailed deer in Canada and Mexico in the 1500s and into the early 1900s. VerCauteren (2003) notes that their range has recently expanded to the north and west (p. 17). The population estimate for the years 2000 to 2016 (black line) is based on data collected from all 50 states as explained by Webb (2018). Rochlin et al. (2022) state that “Deer populations increased beyond precolonial levels.” (p. 415). By measurement, their figure shows a 60% increase in deer density from 1500 to 2020. The consensus among deer researchers is that the population is about the same over that period. Based on the data collected by Webb (2018), about 90% of the U.S. white-tailed deer population is in states that have been home to lone star ticks as illustrated by Figure 1 in Rochlin et al. (2022).

Rochlin et al. (2022) show an exploding white-tailed deer population from 2000 to 2020 (blue boxes), although no sources were cited covering this period. Webb (2018) finds a significant decline from 2000 into 2016 (black line). Using a sample from 29 states, a study for the U.S. Forest Service (Flather et al., 2013) also finds the white-tailed deer population peaking in the year 2000, but with only a modest decline into 2010. Deer population trends vary by state and by region within states. They can significantly change from year to year because of winter weather or water conditions.

Four “data sources” were cited by Rochlin et al. (2022) for their deer density estimate. One from Pierce et al. (2011) is represented as the green line in Figure 1 which shows an estimate for Missouri from 1705 to 1900. It shows an increase in the deer population from 1805 into 1830 attributed to farming activities that improved deer habitat (p. 2). Other states had similar experiences with varying times and impact. McCabe and McCabe (1984) show a modest short upward blip in a downward trend as the human population ramped up. VerCauteren (2003) shows a slowing of the downward population trend represented by the black line in Figure 1.

Deer were nearly extirpated in Missouri by the early 1900s. Restocking was required to restore the herd and the state experienced a more delayed rebound than other states. It is unclear where Rochlin et al. (2022) find their support for the increase in the deer population from 1625 to 1745 (blue boxes in Figure 1). The Missouri data show a deer population decrease from 1705 to 1815 then a short increase to 1830, a short pause in a long-term decline.

The Missouri data do show a 16% increase in the deer population from 1705 into 2000, but the 1705 population begins on a downward trend, indicating a higher previous population. The pattern of decline and recovery varied by state.

Another article cited by Rochlin et al. (2022) discusses the well-documented recovery of the white-tailed deer population from the early 1900s to 2000 (Paddock and Yabsley, 2007) as a result of conservation efforts. Their chart showing the deer population of Arkansas, Georgia, Florida, and Indiana tracks more closely with the black line in Figure 1 than the blue boxes of Rochlin et al. (2022).

A third article cited by Rochlin et al. (2022) contains some general discussion about Pennsylvania and cites McCabe and McCabe (1984) for historical background (Redding, 1995). Redding describes the data in his article as “Actual data for estimates of the deer population taken in Warren, Forest, McKean, and Elk Counties.” His data from the 1930s into the early 1990s show the deer population at about the same levels. His discussion about deer population trends is generally limited to northwestern Pennsylvania where he suggests there was a probable increase in the population during the early 1800s as the result of settlement activities.

The fourth article cited by Rochlin et al. (2022) is a descriptive history of New York (Severinghaus and Brown, 1956). They acknowledge that the consensus is that there were large numbers of deer in the Northeast pre-colonization but speculate that for New York, given the mature forests found by early explorers, “… it appears questionable if deer found more than comparatively small areas of suitable habitat in the vast expanses of virgin timber” (p. 132). Is this type of comment considered to be data? And of course, this is just one state.

The tick population trend (red boxes in Figure 1) from Rochlin et al. (2022) appears to be drawn to conform with the deer density trend in the figure (blue boxes). In comparison to other deer research, the article by Rochlin et al. (2022) overstates the correlation between deer and ticks as characterized in their Figure 3. The increase in the tick populations from 1665 to 1750 shown in their Figure 3 does not appear to be associated with a rising deer population based on other research. The low tick population around 1675 is associated with relatively high deer populations based on other research.

If the tick information in their Figure 3 is approximately true, then perhaps more attention should be given to landscape management and the natural fire cycle, other factors they discuss. However, in their conclusion they warn about the “exploding deer population” (p. 418). This phrase will reinforce a common public misconception about recent changes in the white-tailed deer population, that it is exploding out of control when it has returned approximately to the pre-colonial level.

Unfortunately, the problematic white-tailed deer information provided by Rochlin et al. (2022) muddles the science available to inform tick management public policy. Given the complexity of estimating the historic white-tailed deer population density and the limitations of the four articles cited, does the article meet the standard for publication in a scientific journal? What will the authors do to improve our understanding of these historical relationships with the goal of advancing scientific knowledge?

## References

[CIT0001] Flather, C. H., M. S. Knowles, M. F. Jones, and C. Schilli. 2013. Wildlife population and harvest trends in the United States: a technical document supporting the Forest Service 2010 RPA Assessment. U.S. Department of Agriculture, Forest Service, Rocky Mountain Research Station, Ft. Collins, CO.

[CIT0002] McCabe, R. B., and T.R. McCabe. 1984. Of slings and arrows: an historical retrospection, pp 19–72. In Halls, L.K., (ed.), White-tailed deer: ecology and management. Stackpole Books, Harrisburg, PA.

[CIT0003] Paddock, C. D., and M. J. Yabsley. 2007. Ecological havoc, the rise of white-tailed deer, and the emergence of *Amblyomma americanum*-associated zoonoses in the United States. Curri. Top. Microbiol. Immunol. 315: 289302–289324. 10.1007/978-3-540-70962-6_12 17848069

[CIT0004] Pierce, R. A., J. A. Sumners, and E. Flynn. 2011. Ecology and management of white-tailed deer in Missouri. University of Missouri–Columbia. Extension Division. g9479, Columbia, MO. (https://extension.missouri.edu/publications/g9479).

[CIT0005] Redding, J. 1995. History of deer population trends and forest cutting on the Allegheny National Forest, pp. 214–224. In Gottschalk, Kurt W.; Fosbroke, Sandra LC, (ed.), Proceedings, 10th Central Hardwood Forest Conference; 1995 March 5–8; Morgantown, WV.: Gen. Tech. Rep. NE-197. US Department of Agriculture, Forest Service, Northeastern Forest Experiment Station, Radnor, PA. (https://www.fs.usda.gov/treesearch/pubs/12759).

[CIT0006] Rochlin, I., A. Egizi, and A. Lindström. 2022. The original scientific description of the lone star tick (*Amblyomma americanum*, Acari: Ixodidae) and implications for the species’ past and future geographic distributions. J. Med. Entomol. 59: 412–420. doi:10.1093/jme/tjab215 35024845

[CIT0007] Severinghaus, C. W., and C. P. Brown. 1956. History of the white-tailed deer in New York. N. Y. Fish Game J. 3: 129–167. (https://www.dec.ny.gov/docs/wildlife_pdf/histdeernewyork.pdf).

[CIT0008] University Of Florida. 2000. UF research: does make up for losses of hunted bucks. Science Daily. (https://www.sciencedaily.com/releases/2000/11/001122183103.htm). November 28.

[CIT0009] VerCauteren, K. C. 2003. The deer boom: discussions on population growth and range expansion of the white-tailed deer. USDA National Wildlife Research Center—Staff Publications. 281. (https://digitalcommons.unl.edu/icwdm_usdanwrc/281/).

[CIT0010] Webb, G. K. 2018. Searching the internet to estimate deer population trends in the U.S., California, and Connecticut. Issues Inf Syst. 19(2): 163–173. (http://www.iacis.org/iis/2018/2_iis_2018_163-173.pdf). Data and other information available: https://www.deerfriendly.com/decline-of-deer-populations.

